# A Diagnostic Sign of Cancer

**Published:** 1888-03

**Authors:** A. McNeil


### A DIAGNOSTIC SIGN OF CANCER.

Dr. Rommelaere found that in thirty-four cases of cancer the daily excretion of urates in the urine decreased steadily until it sank to less than twelve grammes per day, while in twelve cases of round ulcer of the stomach the quantity of the urates amounted to twenty-five grammes per day; therefore, in cases in which the diagnosis is uncertain, the decrease of the urates will be decisive.

Dear Doctor:—The above I translate from the *Zeitschrift* which translated it from the *Journal de Medicine de Bruxelles*, and is a valuable point, if reliable, in the diagnosis of cancer, particularly in that of the stomach.

A. McNeil.

